# Comparing Human Video Modeling to Animated Video Modeling for Learners with Autism

**DOI:** 10.1007/s40616-025-00224-y

**Published:** 2025-11-12

**Authors:** Christopher Bloh, Lynn Bacon, Barbara Begel, Katherine Madara, Brianna Koller

**Affiliations:** 1 Department of Special Education, Kutztown University, Kutztown, PA USA; 2 Allentown School District, Allentown, PA USA; 3 Department of Special Education, Kutztown University, Kutztown, PA USA

**Keywords:** Autism, Video modeling, Intraverbal, Motor imitation, Communication, Social skills

## Abstract

People with autism spectrum disorder (ASD) may have difficulty responding vocally with intraverbals and physically with motor imitation during conversations. Not responding with an appropriate word coupled with an absence of body language could compromise social opportunities. The literature lists scores of studies implementing human video modeling to increase skills of people with ASD but not much research has been conducted using animated video modeling (Kellems et al., 2020). This study compared human video modeling to animated videos to teach vocal intraverbal responding along with motor imitations of facial expression and body language to eight children with ASD. Seven of the eight participants acquired the target behaviors with one or both methods to some degree. Two participants demonstrated more of the target behaviors with the human video, three demonstrated more with the animated, and little difference in learning was observed for three participants. One participant only demonstrated target behaviors following the human video and another only demonstrated target behaviors following the animated video, suggesting that both methods could be effective and neither was conclusively superior.

Conversational and social skills can be complex. From a verbal behavior perspective, behaviors of both the speaker and listener can be analyzed and categorized into operants. Social interactions routinely require multiple expectations, as a listener may be required to vocally respond to a speaker with an intraverbal and/or imitate the physical behavior of the speaker with motor imitation. Common examples of intraverbals are responding to others’ questions/prompts and/or filling in blanks prompted by others. Additionally, a listener may demonstrate interest in a speaker’s behavior by physically copying their behaviors or engaging in motor imitation, which is, in turn, reinforced with attention from the speaker.

Video modeling involves a model displaying target behaviors to be imitated by the observer and has been widely implemented to increase intraverbals, motor imitation, and other operants for people with autism spectrum disorder (ASD; Alhuzimi, 2022; Amadi et al., 2022; Bellini & Akullian, 2007; Charlop & Milstein, 1989; Ledoux et al., 2020; Maione & Mirenda, 2006; Mason et al., 2012; Paterson & Arco, 2007). More specifically, it has been used to target intraverbals, such as responding to questions (Dueñas et al., 2021; Charlop & Milstein, 1989; Miltenberger & Charlop 2015; Sherer et al., 2001), to teach motor imitation for abduction-prevention (Bell, 2022), and social play (Briet et al., 2023). Furthermore, video modeling has been found to be more effective than in vivo or live modeling for task acquisition and generalization (Charlop-Christy et al., 2000).

Animated video modeling is a growing area of research targeting behaviors for people with ASD. Atherton and Cross (2018) suggested that an area of social-cognitive strength for people with ASD is their potential ability to respond to the anthropomorphic stimuli of animals or animation (Yamada et al., 2013). Animated modeling has been used to target social engagement through motor imitation (Ho et al., 2019), vocal responding with intraverbals, and eye contact/body posturing though motor imitation (Kellems et al., 2020). However, Kellems and colleagues cited limited research available for animated video modeling targeting social skills. To date, there is little research available comparing human video modeling to animated video modeling in the acquisition of social skills.

Recognizing the evidence for human video modeling to teach social skills (intraverbals, motor imitation) and the potential for using animated video modeling for the same purpose, this study compared the two methods with children with ASD. One human and one animated video were created for each participant, each demonstrating three social interactions. The video model responded to the vocal prompts of an unseen speaker with a vocal response (intraverbal), making a facial expression (motor imitation), and posturing her body (motor imitation). After both videos were shown to participants, the same respective prompts were presented to investigate whether human or animated video differentially occasioned intraverbal and motor imitative responding.

## Methods

### Participants and Settings

Eight children with ASD (ages 5–8) participated in the current study. Half were enrolled in an urban, public early childhood center and half attended a kindergarten to second grade classroom (Table 1). The study took place in self-contained, autistic support classrooms. Sessions were consistently conducted in an unoccupied area within those classrooms. Typical to any classroom, tables, desks, white boards, and computers were present with 6–8 long windows. Participants were included because of their ASD diagnoses and they were not receiving intraverbal or motor imitation instruction outside of this study.

**Table 1:** Demographic Information and Assessment Scores

Participant	Gender	Age	Race/Ethnicity	Diagnosis	EESA	MIS	IV Subtest
Isaac	Boy	7	Caucasian/Latinx	ASD, ADHD	100	32	80
Mark	Boy	5	Latinx	ASD, OHI	0	32	38
Anesha	Girl	7	African American/Latinx	ASD	0	32	0
Maria	Girl	6	Latinx	ASD	0	32	0
Ray	Boy	6	Latinx	ASD	0	30	0
Kadeem	Boy	8	African American	ASD	85	32	0
Leon	Boy	6	Latinx	ASD	0	28	0
Gavin	Boy	7	Latinx	ASD	0	32	0

### Materials

Materials included an iPhone/Android video recorder, iPad, human model recordings, animated model recordings, and assessment instruments. The first author created two social scripts, which were roleplayed with another adult and video recorded. One video was used as the human video. The second video (i.e., the animated video) was created on iMovie (https://www.apple.com/imovie/) using rotoscope animation. Rotoscope animation involves tracing over video footage creating animated sequences. For purposes of brevity, the rotoscope animation is referred to as “animated.” Thus, the same model was used for both videos, wearing the same clothes, and in the same setting. The animated model, taken from the actual human video, appeared drawn and colored to appear “animated.”

### Dependent Variables

Dependent variables were intraverbal or vocal response (VR), motor imitation through facial expression (FE), and body expression (BE). VR was vocally responding, FE was manipulating one’s facial muscles/head positioning, and BE was repositioning part(s) of one’s body to the teacher’s prompts with the operationally defined response within 5s (Tables 2, 3). After viewing a video, the teacher presented the discriminative stimuli displayed in that video. For the intraverbal/VR, a response was considered correct if the participant’s vocal response was the same as the model in the video. For example, the video model responded “sun” when prompted “What is in the sky?” If a participant responded “stars” after the same prompt, i.e., “What is in the sky?” during testing, it was scored an incorrect response, although that response could be an appropriate intraverbal in another setting. Additionally for the FE and BE, responses were scored correct only if they were the same as the model displayed. Each video modeled three different interactions of VR, FE, and BE. Thus, the cumulative number of correct responses were 0–9 for each session (Tables 4 and 5).

**Table 2:** Discriminative Stimuli and target responses for human video modeling

Participant	Discriminative stimuli	VR	FE	BE
Isaac	“Who made the Thinker?”	Rodin	scrunches face	puts fist to chin (wrinkles nose, raises cheeks to eyes)
	“What were yew trees	bows	widens eyes	draws bow
				used for?”
	“King Solomon was …”	wise	smiles	points to temple
Mark	“Peek-a …”	boo	widens eyes	palms facing Clinician
	“What do you read?”	book	looks down	palms upward at chest level
	“Sugar pie honey …”	bunch	head back	fist to mouth
Anesha	“What is in the sky?”	sun	smiles	extends fingers
	“What does a frog do?”	ribbit	closes eyes	hands parallel and flat
	“Mary had a little …”	lamb	tilts head	folds hand and places on cheek
Maria	“Up and …”	down	looks down	points down
	“3-2-1”	blast off	looks up	extends fingers on both hands
Ray	“You drink …”	milk	pursed lips	makes drinking motion
	“See you …”	later	tilts head to side	waves
	“You sleep in a …”	bed	closes eyes	palms together
placed on cheek				
Kadeem	“The weasel goes …”	pop	opens mouth	points forward
	“What’s new pussycat?”	wo	head back	fist to mouth
	“What does a hyena do?”	bites	bares teeth	makes claws
	“Do you like cabbage?”	no	scrunches face	pushes away
Leon	“A kitty goes …”	meow	closes eyes	motions whiskers on face
	“A siren goes …”	woooo	widens eyes	points up
	“A cow goes …”	moo	face down	fists to head like horns
Gavin	“What do we smell with?”	nose	tilts head back	points to nose
	“What do we wear on our feet?”	shoes	tilts head down	points down
	“What does a duck do?”	quack	puckers lips	flaps arms

**Table 3:** Discriminative Stimuli and target responses for animated video modeling

Participant	Discriminative stimuli	VR	FE	BE
Isaac	“Four laps around a track is a …”	mile	closes eyes	running motion
	“Neil Armstrong walked on the …”	moon	eyes towards ceiling	arms extended laterally
	“DaVinci painted the …”	Mona Lisa	pouts	hands under chin
Mark	“What do you do with a spoon?”	eat	opens mouth	lifts fist to mouth
	“What does a hyena do?”	bites	bares teeth	claws forward
	“Pop goes the …”	weasel	turn head to side	thumb up
Anesha	“Peek-a …”	boo	widens eyes	hands to face extending fingers
	“What does a sheep do?”	baaa	bows head to chest	extends arms and ‘walks’
	“Sugar pie honey …”	bunch	smiles	fist to mouth
Maria	“A cow goes …”	moo	closes eyes	fists to head like horns
	“You brush your …”	teeth	bares teeth	fist over mouth
	“A car goes …”	room	tilts head back	extends arms forward
Ray	“What is in the sky?”	sun	widens eyes	extends fingers
	“A cheetah goes …”	roar	head back	arms extended
	“What does an airplane do?”	flies	tilts head down	extends arms at
Kadeem	“What can you eat?”	apple	opens mouth	holds hand to mouth
	“What does a crow do?”	caw	closes eyes	flaps arms
	“What can you drink?”	milk	pursed lips	makes drinking motion
Leon	“Fire is …”	hot	scrunches face	palms extended Forward
	“Let’s do the …”	twist	head back	fists close to body
	“Thunder and …”	rain	looks down	motions down
Gavin	“Peek-a …”	boo	widens eyes	hands to face extending fingers
	“What do we hear with?”	ears	turn head to side	points to ear
	“Up and …”	down	tilts head down	points down

**Table 4:** Participant data beginning with Animated then Human Video

Participant	Animated Video			Human Video		
	Sessions to exit criterion	Generalization	Maintenance	Sessions to exit criterion	Generalization	Maintenance
Isaac	12*	33%	55%	5	33%	11%
Mark	7	100%	100%	6	89%	77%
Anesha	10*	0%	11%	10*	0%	0%
Maria	10*	0%	0%	10*	0%	44%

Asterix represents that participant did not meet mastery criterion for 10 consecutive sessions and that intervention was discontinued.

**Table 5:** Participant data beginning with Human then Animated Video

Participant	Human Video			Animated Video		
	Sessions to exit criterion	Generalization	Maintenance	Sessions to	Generalization	Maintenance
			exit criterion			
Ray	18	66%	66%	10	77%	77%
Kadeem	4	22%	66%	5	44%	44%
Leon	10*	0%	33%	14	88%	77%
Gavin	10*	0%	0%	10*	0%	0%

Asterix represents that participant did not meet mastery criterion for 10 consecutive sessions and that intervention was discontinued.

### Assessments

#### Verbal Behavior Milestone Assessment Intraverbal Assessment Subtest

The Verbal Behavior Milestone Assessment Intraverbal Assessment Subtest (VB-MAPP; Sundberg & Sundberg, 2011) was used to assess participants’ intraverbal responses. This assessment has 80 items over eight increasingly difficult sections, with 10 possible responses per section. A correct/self-corrected response counted as 1, with no/incorrect response counted as 0 (Table 1).

#### Motor Imitation Scale

The Motor Imitation Scale (Stone et al., 1997) was utilized to assess motor movements for imitation. This assessment contains 16 items that assess the ability to physically imitate single-step, motor imitation skills. Cumulative scores range from 0–32. Items were scored as 0 (no identified behavior demonstrated), 1 (purposeful, approximation of identified behavior), or 2 (purposeful display of identified behavior).

#### Early Echoic Skills Assessment (EESA) Subtest

The Early Echoic Skills Assessment (EESA) was used to assess participants’ vocal ability to imitate a speech model (Esch, 2008). The EESA consists of 100 one-point items in five groups that increase in complexity from echoing one-two-three syllable words to vocally imitating prosody in spoken phrases and other contexts. Only two participants achieved a score higher than zero (Table 1). Mand and tact assessments were not completed.

#### The Treatment Accessibility Rating Form-Revised

To evaluate social validity, the Treatment Accessibility Rating Form-Revised (TARF-R; Reimers & Wacker, 1992) was administered to teachers and participants’ caregivers at the conclusion at the study. The TARF-R consists of 20 questions related to treatment acceptability, problem severity, and understanding of interventions and was revised highlighting the relevant interests of either stakeholder. The teachers’ TARF-R had all 20 of the original items, while the parents’ version had 12.

### Experimental Design

An adapted multiple baseline design across video model types was implemented. Baseline phases were randomly assigned, staggered across participants, and continued until there consistently appeared to be no responding or incorrect responding. Generalization probes were conducted across people 1–3 days after mastery criteria was met. Maintenance probes followed mastery criteria performance or discontinuation and were collected three weeks after the conclusion of an intervention. Generalization and Maintenance phases were identical to baseline (Miltenberger & Charlop, 2015).

### Procedures

#### Baseline

The teachers enacted the social scripts without presenting the human or animated videos and recorded participant responses. Length of baseline varied across participants and was collected until there appeared no variations in responding. Teachers engaged the participant with scripted discriminative stimuli and no consequences were presented for either correct or incorrect responses (Figs. 2, 3). Incorrect and/or no responses after 5 s for all dependent variables ended the session (Miltenberger & Charlop, 2015). When no variability occurred for the respective dependent variables, baseline ended and the intervention began.

#### Human and Animated Video Modeling

During intervention, human and animated video models were presented to each participant sequentially. Each video was 40–60 s long displaying scenarios involving the three target behaviors. Each video repeated three times alternating scenarios of the target behaviors, (i.e., modeling scenario 1, 2, 3, then 3, 2, 1, then 2, 1, 3; Charlop et al., 2010). The primary author randomly assigned half the participants to view the human videos first and the other half to view the animated videos first. Implementation of an intervention (i.e., either human video or animated video modeling) began for the targeted scenarios while baseline continued for the scenarios in the other video modeling modality. For example, four participants’ interventions began with the animated model scenarios while baseline conditions continued for the human model scenarios (Tables 2, 3). Conversely, the other four participants’ interventions began the human video model while baseline conditions continued for animated. After mastery or exit criteria was met for the first assigned video type, the second was applied. Mastery criteria were 6/9 or higher correct responses (VR, FE, BE x 3 scenarios) per session, for three consecutive sessions. Kroeger et al. (2007) discontinued their study of two social skills interventions for children with ASD if a participant did not meet mastery criteria after 15 sessions. Owing to limited availability of the participants (absences, no school, etc.), the intervention was discontinued if mastery criteria were not met for 10 consecutive sessions. Generalization and maintenance probes were collected the same way data were during baseline.

### Treatment Integrity and Interobserver Agreement

Treatment integrity was calculated for 100% of trials (Bergmann et al., 2023). The first author observed each trial conducted by the teachers and assessed whether procedures were implemented as scripted, identifying extraneous stimuli, unscripted prompts, etc. Each session had a total of nine, scripted interactions. If any procedures varied from protocol, that trial was scored “no.” Treatment integrity was calculated as the cumulative number of scripted interactions divided by nine (total number of interactions per session) and multiplied by 100. Results between the first author and teachers were added together and divided by 2 to obtain the average. Results were compared for 84% of sessions and treatment integrity was 98.9% across participants (range 0–100%).

Interobserver agreement (IOA) was taken for 83% of sessions. The first author and teachers collected data on dependent variables, combined the number of cumulative (VR, FE, and BE total) responses scored in agreement, divided by the total number of responses, and multiplied by 100. IOA was 99.5% across participants (range 89–100%).

## Results

As depicted in Figs. 2 and 3, no participant responded with any of the operationally defined target behaviors during baseline. Mark, Ray, and Kadeem’s responding met mastery criteria with both the human and animated videos. Mark’s responding met mastery criteria for the animated video in seven sessions and human video in six. Ray’s responding met mastery criteria in 18 sessions with the human video and 10 sessions with the animated video. Kadeem’s responding met mastery criteria rather rapidly for both the human and animated videos in 4–5 sessions. Isaac’s responding met mastery with only the human video in five sessions. His responding did not exceed 6/9 correct responses for any session during the animated video intervention and was discontinued after session 16. Leon’s responding met mastery with the animated video in only 14 sessions. Anesha, Maria, and Gavin’s responding did not meet mastery criteria either with the human or animated videos. Please see Figs. 2 and 3.

Isaac’s VR (intraverbal) responding was higher for both the human and animated videos than FE and BE, while Anesha’s was also higher in VR responding but for only the animated video. Maria responded less with VR compared to FE and BE and there were relatively no responses recorded for Gavin. BE responses appeared slightly higher than FE for some participants.

## Social Validity

According to the TARF-R (Reimers & Wacker, 1992), a score of 1 to a particular item suggested strong negation, 4 was neutral, and 7 was suggestive of strong affirmation. Overall feedback from teachers was affirmative rating the intervention as; very acceptable, reasonable regarding student behaviors (6–7), possible disadvantages implementing the intervention (1), and very likely to make permanent improvements in behavior (7). Parent/caregiver feedback was more varied. Seven of the eight participants’ caregivers returned the modified TARF-R. While ratings ranged with acceptability of treatment (4–7), overall ratings were not consistent in; likely to make permanent improvements (1–7), treatment effectiveness (1–7), and confidence in treatment (4–7).

## Discussion

While some research has used animated videos to target attending, little has been completed for social skills (Kellems et al., 2020). We found that three participants met mastery criteria for both human and animated videos (i.e., Mark, Ray, and Kadeem). Two participants only met mastery criteria for the human video (i.e., Isaac and Maria), and one participant only met mastery criteria for the animated video (i.e., Leon). Gavin did not meet mastery criteria for either video modeling intervention.

Prerequisites for successful responding through video modeling involves attending to stimuli, engaging in immediate and delayed imitation, and overall interest in watching videos (MacDonald et al., 2015). Ho et al. (2019) identified a minimum score of 17 for motor imitation for targeting social skills with children with ASD using video modeling. All participants completed most of the assessment motor imitation items and exceeded this score (Table 1). Isaac and Mark were the only participants to obtain a score on the intraverbal assessment. Mark often engaged in echoics and motor imitation during the videos, which seemed to transfer to intraverbals (from echoics) when tested. His responding met mastery criteria for both types of video modeling interventions, while Isaac’s responding only met mastery criteria for the human video. Isaac responded with, “I don’t know,” which could be an intraverbally correct response outside the study but was an operationally incorrect response. Reinforcement history for responding “I don’t know” could have maintained Isaac’s responding, despite being put on extinction with no consequences for responding. Thus, participant learning histories that may affect acquisition should be considered when identifying target behaviors (Kay et al., 2020). While the video modeling literature cites baseline data, assessments for intraverbals prior to intervention has not been included. Consequently, identifying prerequisites for an intraverbal repertoire can be challenging. De Souza et al. (2018) selected a VB-MAPP intraverbal subtest (Sundberg & Sundberg, 2011) score between 50 and 70 for inclusion to teach advanced intraverbals. Future research could consider a more conservative score for inclusion to teach initial intraverbal acquisition.

Anesha and Maria occasionally engaged in echoics, while Mark routinely engaged in echoics and motor imitation during the videos. Unlike the cited research, some participants in this study who met the minimum score for inclusion did not demonstrate acquisition of motor imitation for FE and BE. While a 1-step motor-immediate imitation assessment was implemented for this study, an assessment for delayed imitation assessment was not and participants were required to first attend then later emit the intraverbal (i.e., VR) and two motor imitations, (i.e., FE and BE). Delayed responding during baseline could be investigated as a potential prerequisite for inclusion in future video modeling investigations during a motor imitation assessment. After demonstrating a motor imitation, for example walking a doll, and prompting the participant to do the same, allow 3 s to pass and prompt him/her to do the same with no modeling. One could assume that the higher the preassessment motor imitative score, the more likely the acquisition, but this was not apparent. Future research could consider other assessments for motor imitation, such as the VB-MAPP (Sundberg, 2008) and the ABLLS-R (Partington, 2006).

Some participants’ responding was visibly different from the first to second interventions, with much more responding occurring in the latter. These could have been examples of carryover effects (Barlow & Hayes, 1979), which is the effect of one intervention on another contiguous one. Since the order of the interventions was counterbalanced across participants, it may not have been that one intervention was more effective but carryover effects. The same could be possible for Isaac, who met mastery criteria only for the second. Future research could minimize carryover effects with a true alternating treatment design where interventions follow one another in an unpredictable fashion. One video could be implemented at a time but alternated randomly across sessions.

While Anesha did not meet the minimum 6/9 responses for any session, she responded during more sessions in her first intervention than second, thus discounting carryover effects. It is possible that the first animated video served as an abolishing operation for the second human intervention due to lack of consequences. Perhaps the videos were not reinforcing enough and the lack of consequences put the responses on extinction. Thus, Anesha’s first video had an abative effect for the second. Weyman and Sy (2018) used a praise preference assessment and cited faster acquisition during enthusiastic praise conditions, relative to neutral and no praise conditions. A similar reinforcer preassessment could be used to evaluate the reinforcing effects of physical expressions and voice fluctuations (as implemented in the videos) over attending and could be employed to bolster control through providing secondary measures.

In addition to the intraverbal and motor imitation, an echoic assessment was conducted prior to intervention but mand and tact repertoires were not assessed. Emergent intraverbal behavior typically begins following the acquisition of mand and tact repertoires for neurotypical children (Sundberg, 2008). Thus, established mand and tact repertoires could make the emergence of intraverbals more likely. Future research could identify potential prerequisites for these operants and use the VB-MAPP (Sundberg, 2008) to identify baseline levels for intervention inclusion.

As previously noted, two participants appeared to have higher VR responding compared to FE and BE, four responded the same comparatively, one responded less with VR, and one only responded once. Considering that intraverbals (VR) are typically acquired later than echoics and motor imitation (i.e., FE, BE; Sundberg & Sundberg, 2011), future research could consider using a denser schedule of reinforcement or more salient consequences for correct VR. A differential outcome procedure could be applied where a successful VR response could be paired with a specific and not a generalizable reinforcer (Trapold, 1970). Whereas FE and BE responses could be paired with praise, VR could result in a specific edible, tangible, etc. Furthermore, depicting the dependent variables being reinforced in the video could aid in acquisition.

Some of the operationally defined correct responses may have been incompatible with behaviors in the participants’ repertoire. For example, some FE responses were defined as baring teeth, scrunching his face, etc. Kadeem smiled during every trial. Thus, those facial expressions required him to discontinue smiling and engage in the operationally defined FE. Although he may have been capable of imitating the FE prompts of the model, his continued smiling made it difficult. Future research could identify target behaviors (both FE and BE) that are not incompatible with a participant’s current behavioral repertoire.

This study did not present consequences for correct responses but did present differential reinforcement in the form of praise for compliance. Although video modeling can be effective without the consequential presentation of reinforcement (Lee et al., 2020), reinforcement (by definition) makes behavior more likely to occur. No programmed consequences were presented during baseline in this study, which differs from the cited research in that they provided reinforcement during baseline to avoid inhibition of correct responding. Although watching videos could be a reinforcing activity, it may not be for children with ASD (Ho et al., 2019). Thus, future research could include reinforcement for correct responding during baseline. Additionally, as each of the three presented discriminative stimuli had nine possible correct responses, future investigations could provide specific-praise for correct responses (i.e., “good job touching your head, closing your eyes,” etc.). In specifically reinforcing an intraverbal, a participant would receive additional instruction relative to facial and body expression behaviors, which would not receive behavior-specific consequences. This could increase the acquisition of intraverbals in future studies quicker than facial and/or body expressions but bias the acquisition because of the specific-praise, which could serve as discriminative stimuli for further trials. Although presenting reinforcement for correct intraverbal responses could be a confound because all correct responses after the initial response that contacted reinforcement could be considered as due to reinforcement rather than the video model, the very first occurrence of the imitative response would be attributed to the video model and not reinforcement.

The participants may have not experienced consistent exposure to the independent variables, as some watched a portion rather than the entire video. Thus, differences in results could be due to a participant experiencing a larger dose of one intervention over another. Ensuring that participants experience an equal dose of both interventions should have been done to rule out discrepancies as plausible interpretation of treatment effects (Blowers et al., 2021). Future research could include procedures that ensure participants watch all of the behaviors in both videos. For example, if a participant looked away from the video, the clinician could prompt him/her to attend (Charlop et al., 2010) and/or reinforce attending by presenting reinforcement at the end of an attended video.

Some of the procedural selections may have affected results. The mastery criteria selected for the current study may have affected overall maintenance of acquired skills (Fuller & Fienup, 2018). We selected mastery criteria of at least 6/9 correct responses for three consecutive sessions; However, related literature has identified criteria of 7/9 or higher for 2–3 consecutive sessions (Charlop et al., 2010; Charlton et al., 2020; Ledoux Galligan et al., 2020).

In addition to noting the occurrences of echoics and motor imitations across participants, future research could collect data on tacts for identifying the stimuli in the video and the (self) echoics for continuing to repeat the target responses until the relevant discriminative stimulus is presented. These data could provide additional context for evaluating their performance during the assessment. By collecting data on these behaviors during sessions (i.e., occurrence or nonoccurrence of tacts and echoics), one may be able to identify if there is a barrier to learning present (i.e., the lack of these behaviors may suggest lack of attending) that is confounding the assessment. Future research could additionally conduct a barriers assessment to identify variables that can affect learning prior to intervention (Sundberg, 2008). The potential presence of these barriers presents a difficulty with interpreting the current results because it is unclear if the lack of reaching the mastery criteria for each type of intervention was related to the properties of the video itself (i.e., the stimuli of either the human or animated video) or due to a deficit of a skill such as attending that may be a prerequisite skill in order to benefit from video model-based instruction.

Potential discrepancies in the difficulty of acquiring the imitative responses modeled is an additional area that warrants control techniques to ensure that difficulty in acquiring responses is equivalent across targets from the same dependent variable class (i.e., VR, FE, and BE behaviors). The difficulty of acquiring targets could be influenced by the similarity of antecedents across VR, FE, and BE, with more similar antecedents, potentially increasing the possibility of commission errors (i.e., emitting a response that was modeled but to the wrong antecedent [e.g., Isaac saying “moon” in response to the antecedent “four laps around a track is a…”]). The difficulty of acquiring responses may also be increased by the similarity of responses across targets (e.g., two BE targets for Gavin comprised pointing down or pointing at his ears). Length of antecedents and number of behaviors modeled for each dependent variable class could also increase the difficulty of acquisition. Differences in length of the speech-verbal responses across targets could also increase difficulty of learning one target over another. Identifying target behaviors from the *same* level from the VB-MAPP (Sundberg & Sundberg, 2011) could address this. These behaviors are sequenced and balanced over three developmental levels (0–18, 18–30, and 30–48 months). VR targets could be clustered on the same level for intraverbals and FE and BE could be clustered on the same level for motor imitation.

Targets were identified with the likelihood that they would not be occasioned in their natural settings. In other words, they were not likely to be taught or reinforced outside of the study, so as to maximize experimental control (Fahmie et al., 2023). While these novel targets were identified to minimize the likelihood of type 1 error, they may not have been socially or ecologically valid. Future research could identify targets that have utility across participant settings and assess ecological validity using Fahmie et al.’s (2023) Ecological Validity Research Planning and Evaluation Tool. Additionally, it is possible that the two videos were not distinct enough for participants to distinguish between. Future efforts could exaggerate the differences between videos.

**Fig. 1:**
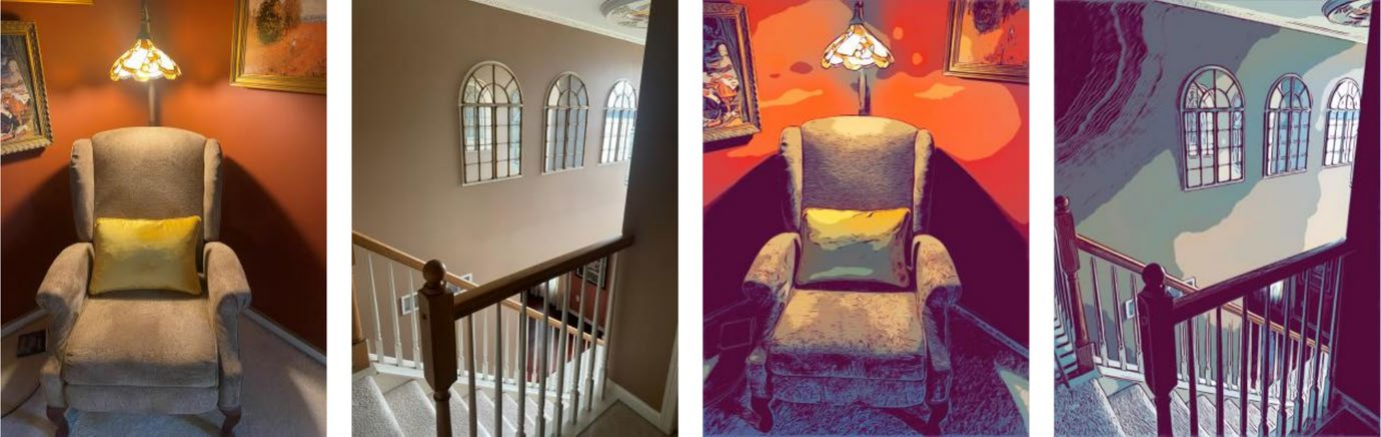
Image stills of video interventions. *Note.* Comparison of actual (human) video modeling stills (left two figures) versus animated video (right two).

**Fig. 2:**
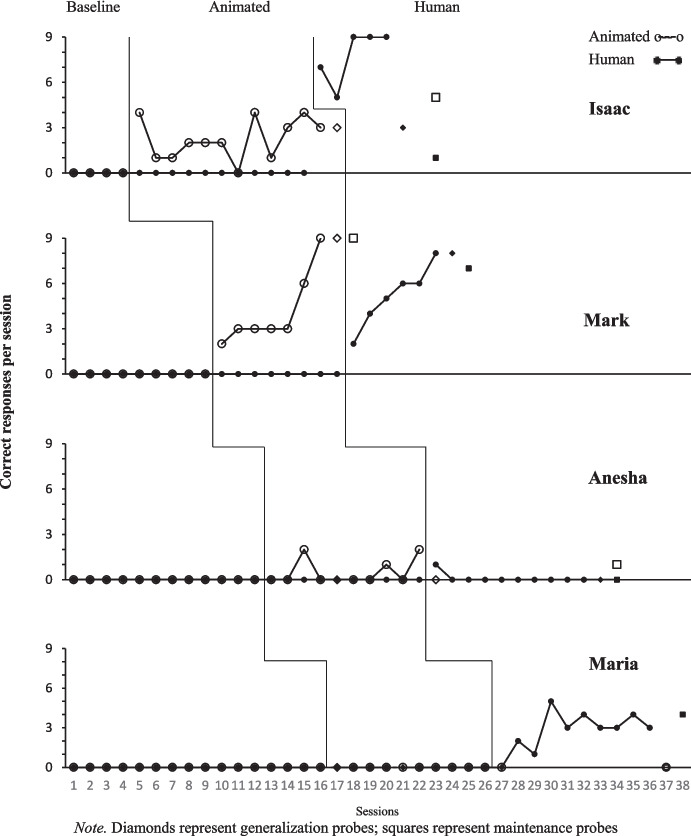
Animated then Human Video Responding for Issac, Mark, Anesha, and Maria. *Note. *Diamonds represent generalization probes; squares represent maintenance probes

**Fig. 3:**
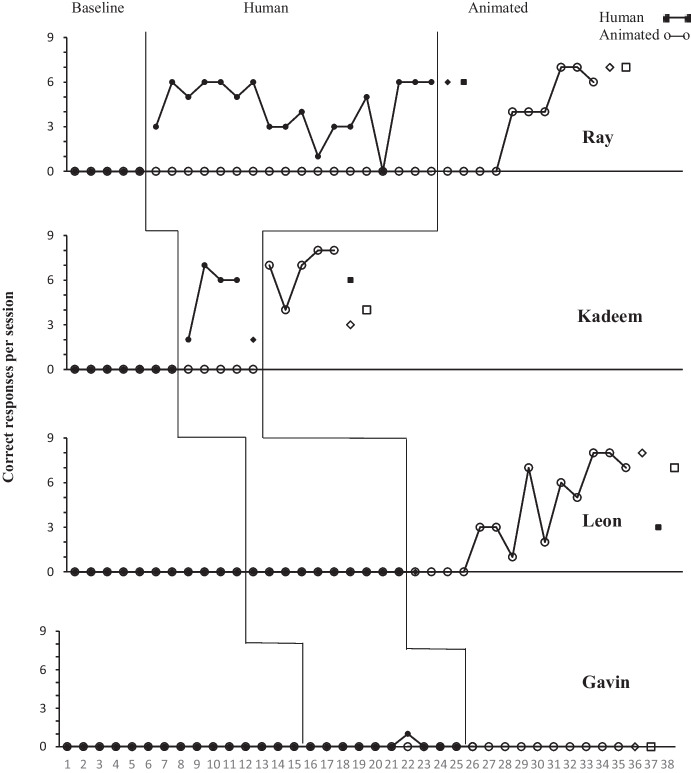
Human then Animated Video Responding for Kadeem, Gavin, Ray, and Leon. Note. Diamonds represent generalization probes; squares represent maintenance probes

In conclusion, it appears that both interventions have utility for clinicians targeting intraverbals and motor imitation used for conversation and social skills with participants with ASD. Additionally, videos can be used both in a telehealth model and through AI. De Knocker and Toolan (2023) found that ASD interventions implemented through telehealth can be beneficial for both caregivers and children. Furthermore, augmentative communication through AI has potential to increase communication and social skills for people with ASD (Wankhede et al., 2024). Both types of videos, once created, could be used countless times for students with similar learning targets. Additionally, scenarios could be cut and pasted to create individualized videos. Video libraries of myriad scenarios could be generated and shown as human or animated videos for different learners Figs. 1, 2 and 3.

## Data Availability

The data generated and analyzed from this study can be made available through correspondence with the first author.
